# Performance of Adaptive Noise Cancellation with Normalized Last-Mean-Square Based on the Signal-to-Noise Ratio of Lung and Heart Sound Separation

**DOI:** 10.1155/2018/9732762

**Published:** 2018-07-12

**Authors:** Noman Q. Al-Naggar, Mohammed H. Al-Udyni

**Affiliations:** Department of Biomedical Engineering, Faculty of Engineering, University of Science and Technology, Sana'a, Yemen

## Abstract

The adaptive algorithm satisfies the present needs on technology for diagnosis biosignals as lung sound signals (LSSs) and accurate techniques for the separation of heart sound signals (HSSs) and other background noise from LSS. This study investigates an improved adaptive noise cancellation (ANC) based on normalized last-mean-square (NLMS) algorithm. The parameters of ANC-NLMS algorithm are the filter length (*L*_*j*_) parameter, which is determined in 2^*n*^ sequence of 2, 4, 8, 16,…, 2048, and the step size (*μ*_*n*_), which is automatically randomly identified using variable *μ*_*n*_ (VSS) optimization. Initially, the algorithm is subjected experimentally to identify the optimal *μ*_*n*_ range that works with 11 *L*_*j*_ values as a specific case. This case is used to study the improved performance of the proposed method based on the signal-to-noise ratio and mean square error. Moreover, the performance is evaluated four times for four *μ*_*n*_ values, each of which with all *L*_*j*_ to obtain the output SNR_out_ matrix (4 × 11). The improvement level is estimated and compared with the SNR_in_ prior to the application of the proposed algorithm and after SNR_outs_. The proposed method achieves high-performance ANC-NLMS algorithm by optimizing VSS when it is close to zero at determining *L*_*j*_, at which the algorithm shows the capability to separate HSS from LSS. Furthermore, the SNR_out_ of normal LSS starts to improve at *L*_*j*_ of 64 and *L*_*j*_ limit of 1024. The SNR_out_ of abnormal LSS starts from a *L*_*j*_ value of 512 to more than 2048 for all determined *μ*_*n*_. Results revealed that the SNR_out_ of the abnormal LSS is small (negative value), whereas that in the normal LSS is large (reaches a positive value). Finally, the designed ANC-NLMS algorithm can separate HSS from LSS. This algorithm can also achieve a good performance by optimizing VSS at the determined 11 *L*_*j*_ values. Additionally, the steps of the proposed method and the obtained SNR_out_ may be used to classify LSS by using a computer.

## 1. Introduction

Lung sound signals (LSSs) exhibit nonperiodicity and low frequency; these signals also contain symptoms of many diseases and interfere with frequency components (50–2500 Hz) with heart sound signal (HSS) frequency in the range of 20–600 Hz [1]. Furthermore, the interference between LSS and HSS is high due to the nearby positions and physiological recording points of the two signal sources. Therefore, the keeping symptoms on LSS overlap and the increase in difficulty of separating HSS and other noise from LSS. They require modern and highly accurate tools for filtering and separation. The adaptive filter (AF) satisfies the LSS purification requirements, and it is an effective tool used to filter LSS from other interference signals or noises. The adaptive noise canceller (ANC) used in this study is a type of AF.

Many works have widely investigated the filtering and separation of LSS by using the ANC or the adaptive line enhancement (ALE) with the last-mean-square (LMS) and normalized last-mean-square (NLMS) algorithms [2–7]. NLMS algorithm can be used to separate HSS from LSS [3, 4] because it can deal with two signals recorded in real time. In general, previous studies have focused on the main parameters of AF, including the filter length (*L*), constant step size (*μ*_*n*_), filter type (such as ALE or ANC), and algorithm (such as NLMS or LMS) to obtain improved AF performance. However, these parameters and combination of techniques have been used with several limitations.

The effect and estimating performance of the designed method were studied using power spectrum density (PSD), which is based on monitoring the concentration of an average power frequency. The PSD graphic shows the comparison before and after signal separation [4–6]. A few studies have also investigated the effects of separating different biosignals from noises, such as LSS, ECG, and myoelectric signals, on the signal-to-noise ratio (SNR) at specified requirement outputs [3, 8–11].

The present study evaluates the estimation performance of ANC based on NLMS algorithm to separate HSS from contaminated LSS on the SNR and the behavior of mean square error (MSE). Moreover, the improvement in performance level is studied under four values of the optimal variable *μ*_*n*_ (VSS) and 11 determined *L*_*j*_ values in the following 2^*n*^ sequence: *j*=2, 4, 8, 16, 32, 64, 128, 256, 512, 1024, 2048. Therefore, the performance for one separation is processed 44 times (4*μ*_*n*_ × 11), that is, the SNR is calculated to obtain 4 × 11 matrix of the output SNR_out_ values. Such combination of the proposed algorithm overcomes the limitations of previous studies in addition to the following: the use of NLMS algorithm instead of LMS algorithm because LMS algorithm cannot be adopted with two long signals and the use of ANC instead of ALE. The VSS initially is studied to identify the optimal range that can work with 11 *L*_*j*_. The level of performance improvement is estimated by comparing the SNR before and after applying the proposed method. The proposed method is carried out and processed using a code program on the MATLAB platform. The proposed method can deal with large data, process repeatedly according to the number of the *L*_*j*_ values, and calculate the SNR_out_ values.

Results revealed the ability of the designed ANC-NLMS algorithm to separate HSS from LSS successfully and showed the increasing performance with increasing *L*_*j*_ value. The improved SNR of the normal and abnormal LSSs is particularly achieved at the *L*_*j*_ range of 64–1024 and 512–2048, respectively, at the determined *μ*_*n*_. The comparison of SNR_in_ with the obtained matrix of the SNR_out_ aids in exploring the existence of distinguishable characteristics between normal and abnormal LSSs, which can be used in computerized LSS classification.

## 2. Materials and Methods

### 2.1. Materials

**Figure 1:**
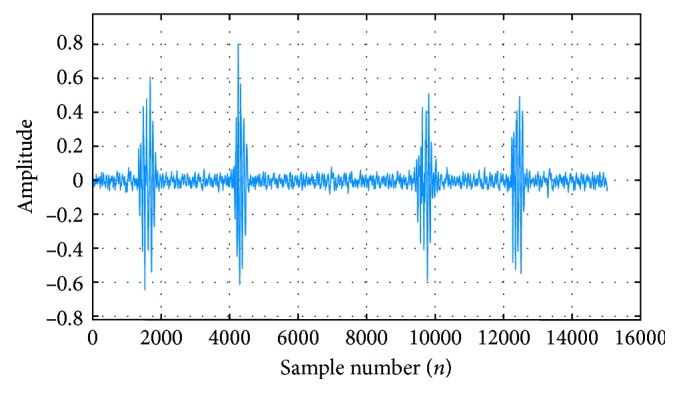
Heart sound signal.

Required signals of heart and lung sounds used for experiments are obtained from the laboratory of the Biomedical Engineering Department at University of Science and Technology, Yemen. LSS and HSS are recorded using two-channel electronic stethoscopes and stored in wav format [4]. Both signals are recorded with sampling frequency of 44100 Hz. HSS recording is carried out on the down anterior region of the chest. The HSS used in this study is normal, which consists of the first heart sound (S1) and murmurs (shown in Figure 1).

The lung sound auscultation is performed on the left down posterior and right anterior regions of the chest. The lung sounds considered in this study are described in Table 1.

### 2.2. ANC Algorithm Architecture

**Figure 2:**
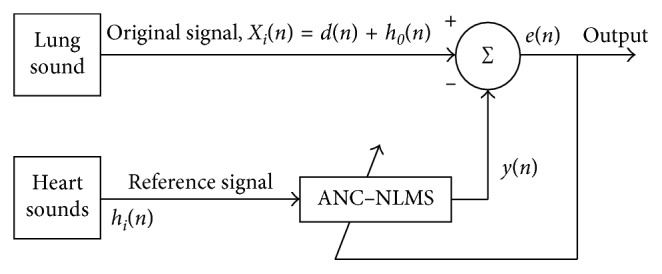
ANC-NLMS algorithm architecture.

The present study investigates on the performance of the combination of ANC with NLMS to separate HSS from LSS. The NLMS is more stable than the LMS in terms of dealing with more than one signal in real-time applications; the NLMS algorithm also displays higher and faster rate of convergence than that of LMS [12]. According to the stated abilities, NLMS algorithm is used in this case study. The original input of ANC is used for contaminated LSS, and the reference input is used for the noise HSS.  Figure 2 illustrates the main components of ANC-NLMS algorithm architecture.

The inputs of ANC-NLMS represent two wave files, each of which is recorded by an individual channel. The original signal *X*_*i*_(*n*) is contaminated by the reference signal *h*_*i*_(*n*) during the recording process.

The original input signal *X*_*i*_(*n*) can be described as follows:

(1) $$\begin{array}{c} X_{i}\left(n\right)=d_{i}\left(n\right)+h_{i0}\left(n\right), \end{array}$$

where *d*_*i*_(*n*) is the desired pure lung sound (LSS), *h*_*i*0_(*n*) is the interfered HSS in *X*_*i*_(*n*) that represents noise, and *i* is a corresponding order number of the signal.

- The reference input signal, that is, *h*_*i*_(*n*), is assumed almost correlated with *h*_*i*0_(*n*).

The filter output *Y*_*i*_(*n*) is defined as follows:

(2) $$\begin{array}{c} Y_{i}\left(n\right)=\overset{L-1}{\underset{k=0}{\sum }}w_{k}\left(n\right)\;*\;X\left(n-k\right)=w^{T}\left(n\right)x\left(n\right)\;\left(\text{estimate of }d\left(n\right)\right), \end{array}$$

where *L*_*j*_ is the filter length, and *j* is the value determined from the 2^*n*^ sequence of 2, 4, 8, 16, 32, 64, 128, 256, 512,1024, 2048, at which the designed algorithm performance is examined. Additionally, *k* is a number of iteration, *x*(*n*)=[*x*(*n*) · *x*(*n* − 1) … *x*(*n* − *L* − 1)]^*T*^ is the input vector of time delayed input values, and *w*(*n*)=[*w*_0_(*n*) · *w*_1_(*n* − 1) … *w*_*L*−1_(*n*)]^*T*^ is the weight vector at the time *n* that can be minimized, as shown in

(3) $$\begin{array}{c} {\left\|w\left(n\right)\right\|}^{2}={\left\|w\left(n+1\right)\cdots w\left(n\right)\right\|}^{2}. \end{array}$$

(ii) The *μ*_*n*_ value for the input vector is calculated as follows:

(4) $$\begin{array}{c} \mu_{n}=\frac{\alpha }{\beta +{\left\|X_{n}\right\|}^{2}}, \end{array}$$

where *β* is a small positive constant used to avoid division by zero when the input vector *X*_*n*_ is zero. Thus, the problem on obtaining a gradient noise amplification in tap weights is solved. Furthermore, *α* is the adaptation positive constant that is commonly less than 1 (0 < *α* < 1) [12, 13].

### 2.3. NLMS Optimization

NLMS optimization is a principal method for minimal disturbance presented in [13], where the error signal *e*_*i*_(*n*) is defined as the difference between the desired signal and the filter output in (2). Hence, the error is minimized in magnitude and rearranged as follows:

(5) $$\begin{array}{c} e_{i}\left(n\right)=d_{i}\left(n\right)-Y_{i}\left(n\right)=d_{i}\left(n\right)-w^{T}\left(n+1\right)x\left(n\right). \end{array}$$

The NLMS algorithm recursion obtains the constrained optimization criterion. The tap weight is as follows:

(6) $$\begin{array}{c} w\left(n+1\right)=w\left(n\right)+\alpha \frac{x\left(n\right)}{\beta +x{\left(n\right)}^{2}}\times e\left(n\right). \end{array}$$

NLMS algorithm is an indication of the minimal disturbance among iterations [13, 14].  Table 2 summarizes the NLMS algorithm.

### 2.4. NLMS *μ*_*n*_

The *μ*_*n*_ parameter should be optimized to ensure the reliability of the designed algorithm [15] at 11 *L*_*j*_ values (determined previously) as a case study. The optimal *μ*_*n*_ is obtained through the following steps. First, most ideal VSS is randomly searched. Results from the first step are used in the second step. Such results include the implementation and automation of the algorithm work. Both steps are described in further detail in the following paragraphs.

- First step: random search for the most ideal possible *μ*_*n*_
- *μ*_*n*_ presents two main parameters, namely, *α* and *β,* which can affect the overall performance of the algorithm. This aspect is the motivation for the VSS approaches, that is, two parameters (*α* and *β*) will be controlled to satisfy the required performance. The experiments are carried out with consideration of the following:
  - The adaptation constant *α* is changed within the range of 0 < *α* < 1, and the small positive constant *β* is changed within the range 0.1–0.009 [12].
  - The VSS is studied within the range of 0‐1 at the determined *L*_*j*_ value.
  - The influence on the overall performance is monitored on the minimization of MSE, SNR_out_ behavior, and algorithm output graphics.
- Second step: auto-optimum VSS
- The proposed idea here is a modified method from pseudorandom number generator *μ*_*n*_ for NLMS algorithm [16]. The main parameters *α* and *β* are changed randomly into variable value from random numbers of distribution from 0 to 1 at each iteration time. *μ*_*n*_ is obtained within a fixed optimal range of 0 ≥ *μ*_*n*_=0.1, which is explored experimentally from the first step. The proposed idea is implemented, as shown in Table 3; it achieves the optimum solution of NLMS in Section 2.3.

### 2.5. Performance Analysis

#### 2.5.1. MSE

MSE is a performance function of AF, and its target is the low MSE value for it to achieve a proper performance [13]. Therefore, the values and graph of this quantity are essential to evaluate the performance of the AF. The formula for MSE is given by the following equation:

(7) $$\begin{array}{c} \text{MSE}\left(n\right)=E\left\{e^{2}\left(n\right)\right\}, \end{array}$$

where *E*[·] denotes the statistical expectation, and *e* is the estimated error of AF. The MSE is calculated for the evolution of AF performance during searching for the optimal VSS, as shown in Table 4.

#### 2.5.2. SNR Evaluation

SNR is used as a metric to estimate the performance of the proposed method, and it is defined as the ratio of the amount of signal to the amount of noise [17]. In the present study, SNR is calculated before and after applying the ANC-NLMS algorithm to compare their values for the same signals at the determining condition. The input SNR (SNR_in_) of the recording signal is measured in amplitudes; thus, SNR_in_ must be squared to be proportional to power, as expressed in (8) [18, 19].

(8) $$\begin{array}{c} {\text{SNR}}_{\text{in}}\left(\text{dB}\right)=10\;\log\;10\frac{E{\left[X_{i}\left(n\right)\right]}^{2}}{E{\left[h_{i}\left(n\right)\right]}^{2}}, \end{array}$$

where *X*_*i*_(*n*) is the original signal defined in (1) and considered the signal, *h*_*i*_(*n*) is the reference signal and considered the noise, and *i* refers to the same number of pair signals. Moreover, *E*(·) denotes operations in calculating the expectation calculation in the time domain. According to the proposed method, (8) is suitable for SNR calculation because *h*_*i*_(*n*) is correlated with existing noise (*h*_0_(*n*)) in *X*_*i*_(*n*).

The output SNR_out_ value after applying ANC-NLMS is given by the following equation:

(9) $$\begin{array}{c} {\text{SNR}}_{\text{out}\left({µ}_{n}\cdot L_{j}\right)}\left(\text{dB}\right)=10\;\log\;10\frac{E{\left[Y_{i}\left(n\right)\right]}^{2}}{E{\left[e_{i}^{0}\left(n\right)\right]}^{2}}, \end{array}$$

where *Y*_*i*_(*n*) is the output (pure LSS) of ANC-NLMS and considered the signal, and *e*_*i*_^0^(*n*) is the estimated error (noise measurement) of ANC-NLMS and considered the noise. The higher output SNR (SNR_out_) than that of SNR_in_ indicates the pureness of the obtained LSS and success of the noise removal and consequently improves the performance of ANC-NLMS. The improvement level is estimated as follows:

(10) $$\begin{array}{c} {\text{SNR}}_{\text{imro}.}\left(\text{dB}\right)={\text{SNR}}_{\text{out}}-{\text{SNR}}_{\text{in}}. \end{array}$$

#### 2.5.3. Output Graphics

Visual graphics are used as metrics in observing the change in input and output graphics. These graphics will illustrate the input signals (original and reference) in two windows and two other windows for output signals (pure LSS and estimated error). Accordingly, the change can be easily observed.

The experiment is carried out using MATLAB platform, in which an algorithm code is designed to obtain the main output signals, their graphic matrix (SNR_out_) (11) SNR_in_, and other input parameters.

(11) $$\begin{array}{c} \left[\begin{array}{ccccc} \underset{\mu_{n}\left(1\right),\ell_{j}=2}{\left[{\text{SNR}}_{\text{out}}\right]} & \underset{\mu_{n}\left(1\right),\ell_{j}=4}{\left[{\text{SNR}}_{\text{out}}\right]} & \underset{\mu_{n}\left(1\right),\ell_{j}=8}{\left[{\text{SNR}}_{\text{out}}\right]} & \cdots & \underset{\mu_{n}\left(1\right),\ell_{j}=2048}{\left[{\text{SNR}}_{\text{out}}\right]}\\ \underset{\mu_{n}\left(2\right),\ell_{j}=2}{\left[{\text{SNR}}_{\text{out}}\right]} & \underset{\mu_{n}\left(2\right),\ell_{j}=4}{\left[{\text{SNR}}_{\text{out}}\right]} & \underset{\mu_{n}\left(2\right),\ell j=8}{\left[{\text{SNR}}_{\text{out}}\right]} & \cdots & \underset{\mu_{n}\left(2\right),\ell_{j}=2048}{\left[{\text{SNR}}_{\text{out}}\right]}\\ \underset{\mu_{n}\left(3\right),\ell_{j}=2}{\left[{\text{SNR}}_{\text{out}}\right]} & \underset{\mu_{n}\left(3\right),\ell_{j}=4}{\left[{\text{SNR}}_{\text{out}}\right]} & \underset{\mu_{n}\left(3\right),\ell_{j}=8}{\left[{\text{SNR}}_{\text{out}}\right]} & \cdots & \underset{\mu_{n}\left(3\right),\ell_{j}=2048}{\left[{\text{SNR}}_{\text{out}}\right]}\\ \underset{\mu_{n}\left(4\right),\ell_{j}=2}{\left[{\text{SNR}}_{\text{out}}\right]} & \underset{\mu_{n}\left(4\right),\ell_{j}=8}{\left[{\text{SNR}}_{\text{out}}\right]} & \underset{\mu_{n}\left(4\right),\ell_{j}=8}{\left[{\text{SNR}}_{\text{out}}\right]} & \cdots & \underset{\mu_{n}\left(4\right),\ell_{j}=2048}{\left[{\text{SNR}}_{\text{out}}\right]} \end{array}\right], \end{array}$$

SNR_out_ is used as one of metrics for the improvement of AF performance during searching for the optimal VSS. The obtained SNR is shown in Table 5.

### 2.6. Experiment Procedures

The experiment procedures are summarized as follows:

- Create the coding program.
- Unite the frequency sampling (8000 Hz).
- The maximum duration of studied signal is 3.5 s, that is, one completed breathing cycle, which is equal to 28000 samples.
- Experimentally identify the optimal *μ*_*n*_ range as stated in Section 2.5.
- Procedure is performed with *μ*_1_ for each *L*_*j*_ value (i.e., 11 times according to the *j* values) to calculate and obtain the SNR_out_ 11 times at each *L*_*j*_ value.
- The procedure is repeated similarly with each *μ*_*n*_ value, that is, four values within the determined optimal *μ*_*n*_ value, to obtain 44 total processing for signal at (*μ*_*n*_ · *L*_*j*_), where *j* = 2, 4, 8, 16, … , 2048. Therefore, SNR_out_ is calculated 44 times and updated with each *μ*_*ni*_ to obtain the matrix shown in (11).
- The experiment is carried out on MATLAB platform, in which an algorithm code is designed to obtain the main output signals and performance analysis tools.

## 3. Results

To obtain reliable results during all procedures, including the searching for the optimal VSS, the number of samples and *L*_*j*_ were considered because of their effects on the performance of the designed algorithm.

**Figure 3:**
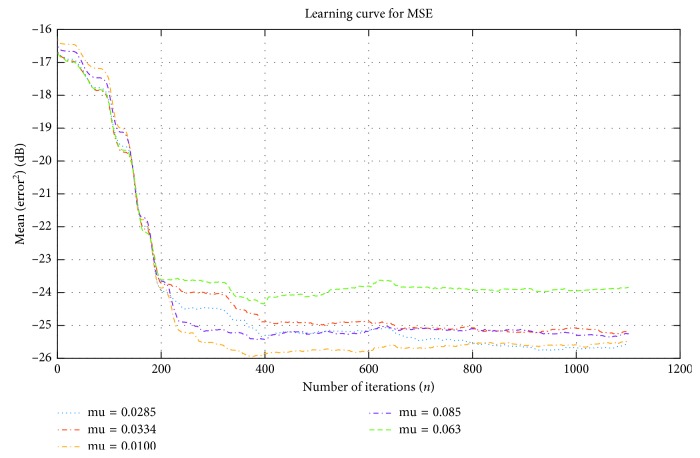
MSE performance of the NLMS for various optimal *μ*_*n*_ values and when *L* = 64.

Figure 3 shows the MSE of *μ*_*n*_ with a value of 0.06, which displays faster convergence rate than those of others. Additionally, the AF became steady after approximately 200 iterations at steady state error of approximately −24 dB. The other MSE tools needed a long time to converge and became steady after approximately 400 iterations at steady state errors of approximately −26 dB for *μ*_*n*_ = 0.033 and −25.5 dB for *μ*_*n*_ = 0.028 and 0.0085. Thus, the steady state errors were small.

**Figure 4:**
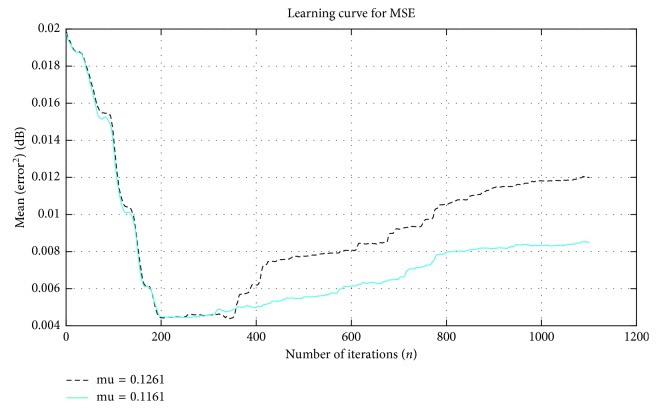
MSE performance of NLMS for *μ*_*n*_ > 0.100 values and *L* = 64.

Figure 4 illustrates the results for large VSS that results in unstable performance and unsatisfied results. According to the comparison between Figures 3 and 4, the performance was good when VSS was small and close to zero. The same conclusion was observed in the changes in MSE and SNR_out_ values; they improved gradually with decreasing *μ*_*n*_ and when they became close to zero, as shown in Tables 4 and 5.

Searching for the optimal VSS identified the VSS optimal range of 0 ≥ *μ*_*n*_=0.1. Thus, the designed algorithm lost its stability when *μ*_*n*_ was used without the identified range, as shown in Tables 4 and 5 and Figures 3 and 4.

**Figure 5:**
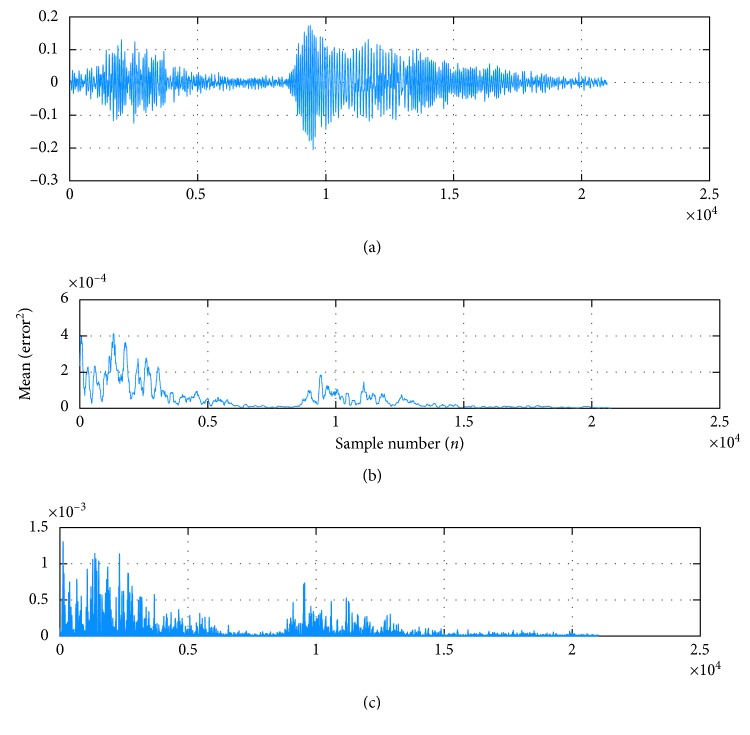
Comparison of the amplitude of AF original signal *X*_*i*_(*n*) and obtained errors. (a) Original signal. (b) MSE of the NLMS in the sample when *μ*_*n*_ = 0.09 and *L* = 128. (c). Output error of the NLMS.

Figures 5(a)–5(c) display that the maximum amplitude of input signal (Figure 5(a)) is 0.18, the maximum amplitude of the error signal is approximately 0.0015 (Figure 5(c)), and the maximum amplitude of square error is 0.00004 (Figure 5(b)). This result showed an increase in the algorithm number of computation windows and minimization of error, as well as MSE, which is considered a function of NLMS performance. Therefore, NLMS optimization achieved minimal disturbance, and the designed algorithm accurately adapted and converged to separate HSS from the original signal (Section 2.3).

The auto-optimal algorithm of identifying VSS has been used to evaluate the separation of HSS from the original signal that consists of contaminated LSS and HSS based on SNR, as well as the performance of ANC and NLMS algorithm combination.

Table 6 shows the SNR_out_ values calculated from the AF outputs for the abnormal LSS case. Results of the comparison of the SNR_in_ values shown in Table 1 and the SNR_out_ values shown in Table 6 indicated that the SNR_in_ values were located from −14.4 dB to −53 dB in an abnormal case. This result suggested that the abnormal LSS included high amount of noise, and the SNR_out_ values changed according to the *L*_*j*_ and determined VSS.

**Figure 6:**
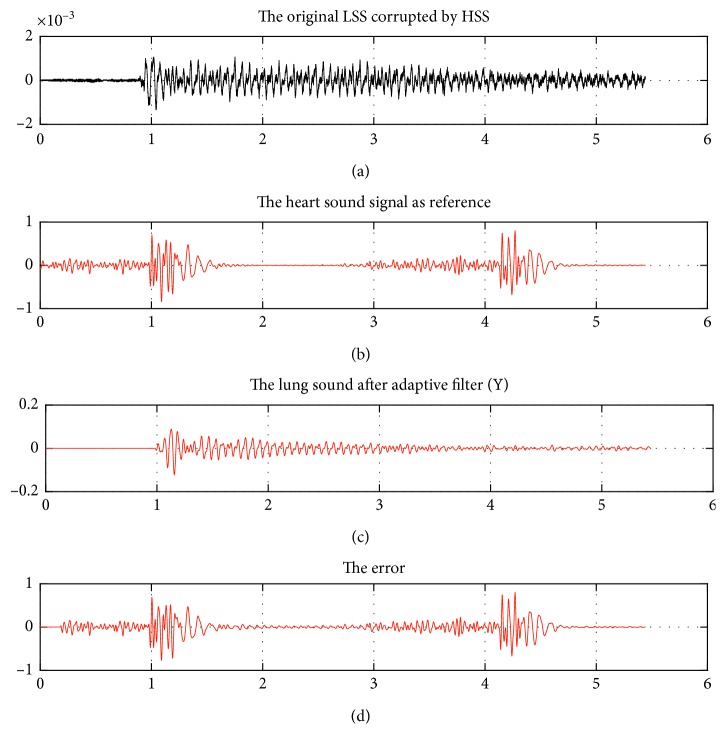
Input and output signal graphics of ANC-NLMS algorithm on an abnormal case (LSA6). Obtained graphics and SNR_out_=−40.58 at *μ*_*n*_ = 0.039 and *L* = 2048, where the input SNR is −53.86 dB.

Figure 6 demonstrates the visual difference before and after applying the proposed method at the determined parameters where the original signal (graphic 1, Figure 6(a)) showed higher frequency components than that of pure LSS (graphic 3, Figure 6(c)). In addition to the improved level of SNR_out_, these results indicated the separation of the noise components from the desired LSS.

Table 7 shows the improved performance level of separating signals on normal LSS case by observing SNR_out_ that started from *L*_*j*_=64 and increased with increased *L*_*j*_ value. The improvement level based on SNR_out_ also increased with increased *L*_*j*_ value and obtained small change at different VSS.

The SNR_in_ values of normal LSS ranged from −3.93 dB to −7.78 dB (Table 1). The SNR_out_ values changed according to the *L*_*j*_ and determined *μ*_*n*_ (Table 7).

**Figure 7:**
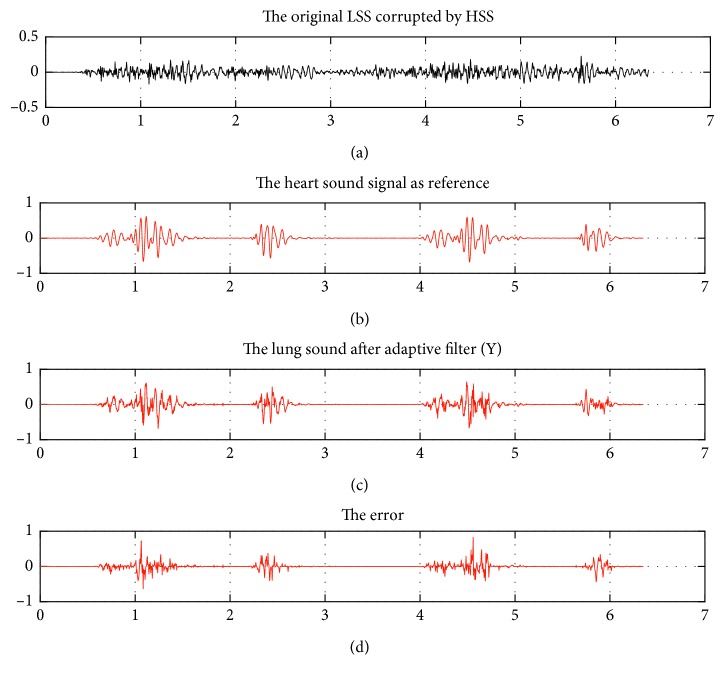
Input and output signal graphics of ANC-NLMS algorithm on a normal case (LSN1). The obtained graphics and SNR_out_ at *μ*_*n*_ = 0.017, where the SNR_in_ is −8.65 dB, and the SNR_out_ is −3.34 dB at *L* = 128.

Figure 7 exhibits the signal obtained by the designed algorithm, with the visual difference between the original signal (graphic 1, Figure 7(a)) and the pure LSS (graphic 3, Figure 7(c)). In general, the frequency components were low in the subjected original signal and decreased after applying AF on the pure LSS (graphic 3, Figure 7(c)).

## 4. Discussions

The optimized results of the designed algorithm determined the optimal VSS range of 0 ≥ *μ*_*n*_=0.1; in this range, the AF became highly stable with nonstationary biosignals, such LSS, where the performance of the proposed method showed the most ideal trade-off between convergence speed and low steady error on the basis of the appropriately autoselected *µ* [7, 15]. This achievement approved the proper work of the designed algorithm and its capability to separate signals by identifying the VSS range on NLMS algorithm, which is in agreement with the results of several works [20–22].

The SNR_out_ values improved progressively at determined *μ*_*n*_ and *L*_*j*_ values for abnormal and normal lung sounds, as shown in Tables 6 and 7, respectively. The SNR_out_ matrix indicated that the performance level in the normal and abnormal LSSs started improving from the *L*_*j*_ values of 64,128 and 256,512, respectively. Moreover, *L*_*j*=1024_ can be the upper limit at which the AF may work stably with normal LSS and obtain accurate outputs. AF can work at *L* more than 2048 with abnormal LSS. This result can be due to that it consists of normal LSS for low-frequency components, and the abnormal LSS consists of high-frequency components.

The SNR_out_ values revealed several distinct markers between LSSs; the normal LSS shows high SNR values, that is, the SNR value reaches close to 0 or the positive axis. By contrast, the SNR values of the abnormal LSS are considerably small in the negative axis. Therefore, these characteristics may reveal clear difference that can differentiate between both LSS types whether in terms of SNR_in_ or SNR_out_ values.

The performance estimation of ANC-NLMS algorithm combination based on automatic identification of the optimal VSS validated the correctness of the proposed method and its sequence steps in separating HSS from LSS. Additionally, such estimation explored the distinct features differentiating normal LSS from abnormal LSS, and these characteristic may be used as primary features to classify LSS.

## 5. Conclusions

This study investigated an effective method of ANC-NLMS algorithm based on automatic identification of the optimal VSS for 11 *L*_*j*_ values to separate HSS from LSS. The performance of the designed algorithm evaluated at determined conditions showed good result by reducing and minimizing the error gradually to zero after the convergence time.

The effectiveness of the designed algorithm to separate HSS from contaminated LSS estimated based on the SNR_out_ illustrated a progressive performance improvement level with increasing *L*_*j*_ and significantly improved separation of HSS from LSS.

This SNR_out_ explored a novel method to differentiate between normal and abnormal LSSs. This method may be used as basis in developing computerized diagnosis and automating LSS calcification.

The proposed approach clarified the correctness of the combined designed algorithm and achieved significant performance. The proposed method may be subject for further study on LSS under different settings and durations.

## Figures and Tables

**Table 1:** Lung sound data.

N	Name	Type of sound	Status	Recording position	SNR_in_(db)
1	LSN1	Vesicular	Normal	Posterior: left, low	−8.65
2	LSA2	Crackles	Abnormal	Posterior: left, middle	−14.4
3	LSN3	Bronchial	Normal	Chest: right, up	−3.93
4	LSA4	Wheeze	Abnormal	Posterior: left, middle	−15.9
5	LSN5	Broncho-vesicular	Normal	Posterior: left up	−7.78
6	LSA6	Crackles	Abnormal	Posterior: right, low	−53.8

**Table 2:** Summary of the NLMS algorithm.

Input:	Tap-weight vector, *w*(*n*),
	Input vector, *x*(*n*), and desired output, *d*(*n*)
Output:	Filter output, *y*(*n*), tap-weight vector update, *w*(*n* + 1)

1. Filtering output signal:	*y*(*n*)=*w*^*T*^(*n*)*x*(*n*)
2. Error estimation:	*e*(*n*)=*d*(*n*) − *y*(*n*)
3. Tap weight and step size parameters adaptation:	*w*(*n*+1)=*w*(*n*)+*α*(*x*(*n*)/*β*+*x*(*n*)^2^) × *e*(*n*)

**Table 3:** Summary of optimum ANC-NLMS algorithm for HSS cancellation.

For time index, *n* = 1, 2,…, *L* filter length *L* (number of iteration) and *j* = [2, 4, 8,…, 2048]	
Input	The number of *L* value (*j*), *N* (1, … , 4) the number of step size
	Tap-weight vector, *w*(*n*),
	Input vector, *x*(*n*)
	Desired output, *d*(*n*)
	Alpha = rand(1, *N*)
	Beta = rand(1, *N*)
Output	Filter output, *y*(*n*)
	Tap-weight vector update, *w*(*n* + 1)

1. Filtering	*y*(*n*)=*w*^*T*^(*n*)*x*(*n*)
2. Error estimation	*e*(*n*)=*d*(*n*) − *y*(*n*)
3. Step size calculation	For *i* = 0 : *L* − 1
	For *j* = 0: *N* − 1
	*m*(*j*) = mu/(*x*(*n*)^2 + *be*)
	If *m*(*j*) > mu max
	*m*(*j*) = mu max
	If *m*(*j*) < mu min
	*m*(*j*) = mu min
	End
	End
4. Tap weight and step size parameters adaptation	*w*(*n*+1)=*w*(*n*)+*μ*(*x*(*n*)/*β*+*x*(*n*)^2^) × *e*(*n*)

**Table 4:** Calculated MSE during searching for the optimal VSS.

#	*µ*	MSE					
		*L* = 4	16	64	128	256	1024
1	0.6	0.00003	Inf	NaN	NaN	NaN	NaN
2	0.2	0.00210	0.00679	NaN	NaN	NaN	NaN
3	0.1	0.00329	0.00329	Inf	NaN	NaN	NaN
4	0.09	0.00336	0.00590	2052.75	Inf	NaN	NaN
5	0.041	0.00347	0.00500	0.00490	0.00864	Inf	NaN
6	0.011	0.00291	0.00155	0.00865	0.00423	0.00475	0.01501
7	0.009	0.00278	0.00028	0.00150	0.00371	0.00413	0.01327

**Table 5:** A sample of searching the optimal *µ*_*n*_ for the designed algorithm (SNR_in_ = −7.78).

#	*µ*	SNR_out_ (dB)									
		*L* = 4	8	16	32	64	128	256	512	1024	2048
1	0.8	−5.3	−3.4	−1.8	−0.4	2.1	0.0	NaN	NaN	NaN	NaN
2	0.6	−6.1	−4.1	−2.4	−0.9	1.4	0.0	NaN	NaN	NaN	NaN
3	0.35	−8.0	−5.7	−3.8	−2.2	−0.1	3.0	0.0	NaN	NaN	NaN
4	0.26	−9.1	−6.6	−4.6	−2.9	−0.9	2.3	6.3	NaN	NaN	NaN
5	0.178	−10.7	−8.0	−5.7	−3.9	−2.0	1.1	5.5	7.5	NaN	NaN
6	0.09	−13.5	−10.7	−8.1	−5.8	−3.9	−0.9	3.6	6.4	7.6	NaN
7	0.0797	−13.9	−11.1	−8.4	−6.2	−4.2	−1.2	3.3	6.2	7.5	0.0
8	0.0088	−24.5	−20.9	−17.6	−14.6	−11.8	−8.8	−5.0	−1.4	1.6	3.8

**Table 6:** SNR after applying the designed algorithm for an abnormal LSS case.

LSS#	*µ*_*n*_	*L*_*j*_ values										
		2	4	8	16	32	64	128	256	512	1024	2048
		SNR_out_ (dB)										
LSA2	0.111	−45.43	−38.31	−34.66	−16.16	−24.08	−19.57	−15.43	−15.09	−11.04	−5.06	−1.05
	0.036	−45.43	−40.01	−32.09	−29.44	−13.39	−20.63	−16.99	−15.34	−3.65	−7.40	−2.16
	0.022	−28.12	−40.01	−32.75	−29.44	−24.66	−18.93	−13.87	−10.41	−3.41	−2.84	−3.08
	0.016	−45.43	−40.01	−19.56	−19.31	−24.66	−17.19	−16.99	−15.34	−11.04	−7.95	−3.95

LSA4	0.037	−35.28	−42.26	−40.03	−34.64	−16.15	−24.17	−17.89	−15.35	−14.04	−11.12	−3.80
	0.028	−51.46	−45.66	−37.56	−34.64	−26.35	−24.40	−11.74	−13.11	−13.46	−11.12	−4.45
	0.025	−51.46	−44.18	−39.50	−31.80	−29.32	−13.80	−19.17	−18.29	−14.04	−3.80	−4.79
	0.010	−37.72	−41.70	−40.03	−34.64	−16.15	−23.30	−20.41	−18.29	−6.95	−7.40	−8.00

LSA6	0.100	−106.11	−101.49	−95.90	−83.13	−82.24	−79.34	−75.47	−56.85	−42.00	−43.41	−32.31
	0.039	−101.38	−113.39	−89.27	−83.13	−86.10	−83.84	−65.40	−48.74	−41.56	−36.56	−40.06
	0.037	−101.38	−115.35	−109.27	−103.13	−89.80	−87.29	−82.06	−58.56	−57.56	−47.07	−40.58
	0.017	−102.41	−95.35	−95.59	−100.87	−80.84	−70.46	−64.75	−48.74	−57.63	−36.11	−46.87

**Table 7:** SNR after applying the designed algorithm on normal LSS.

LSS#	*µ*_*n*_	*L*_*j*_ values										
		2	4	8	16	32	64	128	256	512	1024	2048
		SNR_out_ (dB)										
LSN1	0.064	−24.82	−16.36	−16.82	−13.63	−11.06	−8.52	−6.18	−2.24	0.83	3.76	17.64
	0.021	−24.82	−20.59	−16.82	−13.63	−11.06	−6.51	−7.00	−2.24	2.86	3.58	12.39
	0.017	−24.82	−20.59	−16.82	−8.11	−10.66	−8.18	−3.34	−0.93	3.52	3.73	11.25
	0.010	−24.75	−20.59	−16.82	−11.11	−10.85	−9.20	−7.00	−2.24	3.46	3.58	8.83

LSN3	0.025	−20.90	−18.84	−16.76	−14.79	−13.37	−9.60	−9.49	−8.26	−3.23	−3.68	−0.63
	0.011	−21.17	−16.92	−16.76	−13.31	−12.31	−12.28	−9.49	−2.63	−4.94	−3.68	−1.64
	0.010	−21.17	−17.52	−16.76	−14.79	−13.42	−12.46	−4.64	−5.35	−5.81	−3.68	−1.42
	0.009	−21.17	−18.84	−16.68	−14.79	−13.37	−11.90	−8.21	−2.68	−5.81	−3.68	−1.95

LSN5	0.026	−25.95	−24.07	−19.72	−17.32	−9.96	−10.56	−8.47	−4.76	−0.14	1.78	6.56
	0.020	−24.56	−13.45	−19.11	−12.52	−12.21	−10.51	−8.47	−4.76	2.15	5.02	6.02
	0.013	−27.94	−21.41	−18.37	−17.32	−14.24	−10.29	−8.47	−4.76	−0.78	1.78	4.88
	0.010	−26.35	−19.43	−18.55	−17.32	−9.71	−8.15	−8.47	−4.76	−1.10	1.78	3.94

## Data Availability

The data used to support the findings of this study are available from the corresponding author upon request.
